# Insulin-like growth factor-1-mediated regulation of miR-193a expression promotes the migration and proliferation of c-kit-positive mouse cardiac stem cells

**DOI:** 10.1186/s13287-017-0762-4

**Published:** 2018-02-21

**Authors:** Yuning Sun, Rongfeng Xu, Jia Huang, Yuyu Yao, Xiaodong Pan, Zhongpu Chen, Genshan Ma

**Affiliations:** Department of Cardiology, Zhongda Hospital, Medical School of Southeast University, DingjiaQiao No. 87, Hunan Road, Nanjing, 210009 Jiangsu China

**Keywords:** IGF-1, DNMTs, CSCs, c-kit, migration, proliferation

## Abstract

**Background:**

C-kit-positive cardiac stem cells (CSCs) have been shown to be a promising candidate treatment for myocardial infarction and heart failure. Insulin-like growth factor (IGF)-1 is an anabolic growth hormone that regulates cellular proliferation, differentiation, senescence, and death in various tissues. Although IGF-1 promotes the migration and proliferation of c-kit-positive mouse CSCs, the underlying mechanism remains unclear.

**Methods:**

Cells were isolated from adult mouse hearts, and c-kit-positive CSCs were separated using magnetic beads. The cells were cultured with or without IGF-1, and c-kit expression was measured by Western blotting. IGF-1 induced CSC proliferation and migration, as measured through Cell Counting Kit-8 (CCK-8) and Transwell assays, respectively. The miR-193a expression was measured by quantitative real-time PCR (qPCR) assays.

**Results:**

IGF-1 enhanced c-kit expression in c-kit-positive CSCs. The activities of the phosphoinositol 3-kinase (PI3K)/AKT signaling pathway and DNA methyltransferases (DNMTs) were enhanced, and their respective inhibitors LY294002 and 5-azacytidine (5-AZA) blunted c-kit expression. Based on the results of quantitative real-time PCR (qPCR) assays, the expression of miR-193a, which is embedded in a CpG island, was down-regulated in the IGF-1-stimulated group and negatively correlated with c-kit expression, whereas c-kit-positive CSCs infected with lentivirus carrying micro-RNA193a displayed reduced c-kit expression, migration and proliferation.

**Conclusions:**

IGF-1 upregulated c-kit expression in c-kit-positive CSCs resulting in enhanced CSC proliferation and migration by activating the PI3K/AKT/DNMT signaling pathway to epigenetically silence miR-193a, which negatively modifies the c-kit expression level.

**Electronic supplementary material:**

The online version of this article (10.1186/s13287-017-0762-4) contains supplementary material, which is available to authorized users.

## Background

According to the latest update from the American Heart Association [1], cardiovascular disease (CVD) leads to enormous health and economic burdens worldwide. Unfortunately, the high mortality of patients with CVD is unlikely to be improved over a short time span [2]. Despite the rapid development of diagnostic equipment and drug therapy for CVD, the heart has a weak regeneration ability and only compensates for a variety of CVDs caused by the loss of a large number of myocardial cells through adverse remodeling, eventually reaching the decompensation stage. Although a growing number of recent findings [3–5] have challenged the concept that the heart is a terminally differentiated organ without the capacity to regenerate, cardiac-resident stem cells have been shown to play an important role in cardiomyocyte renewal under both pathological and physiological conditions.

C-kit-positive resident cardiac stem cells (CSCs), which have been shown to differentiate into endothelial cells, smooth muscle cells, and even cardiomyocytes, have been used for cardiac repair [3–5]. CSC migration and amplification have been shown to play roles in cardiac repair. In addition, c-kit activation enhances the migration, proliferation, and survival of CSCs [6].

Insulin-like growth factor (IGF)-1, an anabolic growth hormone, is produced in the liver and regulates cellular proliferation, differentiation, senescence, and death in various tissues [7, 8]. Intriguingly, Johnson and Kartha [9] reported that IGF-1 positively regulated CSC clonogenicity, as indicated by an increased percentage of cells expressing the c-kit receptor. In addition, the phosphoinositol 3-kinase (PI3K)/AKT-1 signaling pathway is important in regulating the effects of IGF-1 in improving infarcted heart function [10].

MicroRNAs are highly conserved in both the intracellular and extracellular environment, and have been reported to modulate biological processes such as cell proliferation, apoptosis, tumor suppressor gene expression, and clinical diagnoses, and even to regulate stem cells [11–13]. MiR-193a has been shown to function as a methylation-silenced tumor suppressor in acute myeloid leukemia that represses c-kit expression [14]. However, the mechanism underlying this regulatory action of IGF-1 on c-kit-positive resident CSCs through miR-193a remains unknown.

Here, we hypothesize that IGF-1 might not only regulate CSC proliferation but also promote the survival and migration of these cells. The PI3K/AKT-1 signaling pathway might also participate in these processes.

Thus, we aimed to clarify the underlying mechanism by which IGF-1 regulates c-kit-positive CSCs, and especially how miR-193a engaged in this process.

### Ethics statement

All animal protocols performed in this study were approved by the Institutional Animal Care and Use Committee of Southeast University, and the procedures were conducted in compliance with the National Institutes of Health Guidelines (approval ID: SYXK-2011.3923).

## Methods

C-kit-positive murine CSCs were isolated using our previously described methods [15]. Briefly, CSCs were obtained from the hearts of 2-month-old wild-type male C57BL/6 mice (Yangzhou Laboratory Animal Center). After the CSCs were cultured for 1 to 2 weeks, a layer of fibroblast-like cells migrated from the adherent myocardial tissue. Some small, round, phase-bright cells emerged from these fibroblast-like cells and were collected using the digestion enzyme Accutase (Millipore, USA). Enriched c-kit-positive cells were further isolated using c-kit magnetic-activated cell sorting (MACS) magnetic beads (Miltenyi Biotec Inc., Germany) according to the manufacturer’s instructions. These separated cells were seeded on poly-d-lysine-coated (Sigma, USA) dishes at a density of 2 × 10^4^ cells/ml in cardiosphere growth medium (CGM: 65% Dulbecco’s modified Eagle’s medium (DMEM)-F12 (HyClone, USA) containing 10% fetal calf serum (Gibco, USA), 2 mmol/l l-glutamine (Gibco, USA), 0.1 mmol/l 2-mercaptoethanol (Sigma, USA), 2% B27 (Gibco, USA), 5 ng/ml basic fibroblast growth factor (bFGF; R&D Systems, USA), 10 ng/ml epidermal growth factor (EGF; Peprotech, USA), 40 nmol/l cardiotrophin-1 (Peprotech, USA), 1 unit/ml thrombin (Sigma, USA), 100 U/ml penicillin G (Gibco, USA), and 100 mg/ml streptomycin (Gibco, USA)). The culture medium was changed every 3 days.

### Characterization of CSCs

C-kit-positive cells were isolated from the expanded cells using MACS, as described above. We performed a flow cytometry analysis to analyze the stem cell phenotypes. CSCs were isolated by enzymatic digestion using accutase, re-suspended in phosphate-buffered saline (PBS) and blocked with 3% fetal bovine serum for 15 min prior to the flow cytometry analysis. A nonspecific mouse IgG1 antibody (BD Biosciences, USA) was used as a control. Subsequently, CSCs were labeled with FITC-conjugated rat anti-mouse c-kit, FITC-conjugated rat anti-mouse spinocerebellar ataxia type 1 (Sca-1; BD Biosciences, USA), FITC-conjugated rat anti-mouse CD34 (Miltenyi Biotec Inc., Germany) and FITC-conjugated rat anti-mouse CD45 (Miltenyi Biotec Inc., Germany) antibodies for 30 min at 4 °C in the dark, washed twice with cold PBS, and then re-suspended in buffer. The data were obtained with a FACSCalibur flow cytometer (BD Biosciences, USA) and analyzed using WinMDI software.

### Assessment of cell viability

Cell proliferation was assessed by a Cell Counting Kit-8 (CCK-8) assay (Dojindo, Japan). The experimental procedures described below were performed according to the manufacturer’s instructions. Cells were plated in 96-well culture plates at a density of 5–10 × 10^3^ cells/well. Adhesion was verified approximately 12 h later, and the cells were treated with 0 or 100 ng/ml IGF-1 (Sigma) for 72 h. Next, 10 μl CCK-8 solution was added to each well, and the cells were incubated at 37 °C for 1–4 h. The optical density (OD) of each well was then measured at a wavelength of 450 nm using a microplate reader (Bio-Rad Model 550, CA, USA).

### Assessment of cell migration

CSCs were plated in the upper chambers of Transwell plates (8.0 μm pore size; Millipore, Billerica) at a density of 2 × 10^5^ cells/well in 100 μl medium to analyze cell migration. Migration to the lower chamber, which was filled with 600 μl medium, was induced by adding 100 ng/ml IGF-1 to the cells. After 48 h of incubation at 37 °C in 5% CO_2_, the cells were fixed with 4% paraformaldehyde and stained with 0.1% crystal violet to determine the number of cells that had migrated to the bottom side of the filter. The migrated cells were photographed using a phase contrast microscope, and the number of migrated cells was manually counted in three random fields per filter.

### Western blot analysis

Treated cells were washed twice with cold PBS and incubated with lysis buffer (50 mM Tris (pH 8.0), 150 mM NaCl, 0.02% sodium azide, 0.2% SDS, 100 μg/ml phenylmethylsulfonylfluoride (PMSF; Sigma, USA), 50 μl/ml aprotinin, 1% octylphenoxypolyethoxyethanol 630, 100 mM NaF, 0.5% sodium deoxycholate, 0.5 mM EDTA, and 0.1 mM ethylene glycol tetraacetic acid). The protein concentration was quantified using a bicinchoninic acid (BCA) assay kit (Pierce, USA). The proteins were separated by SDS-PAGE (Biosharp, China) and then transferred to polyvinylidene difluoride (PVDF) membranes that were blocked with TBST solution (10 mM Tris-HCl, 150 mM NaCl, and 0.05% Tween-20) containing 5% skimmed milk for 3–4 h at room temperature. The membranes were then incubated with primary antibodies (Abcam, 1:1000 dilution) overnight at 4 °C and then washed three times with TBST for 15 min each wash. Subsequently, the membranes were incubated with a horseradish peroxidase-conjugated secondary antibody (Santa Cruz Biotechnology, 1:5000 dilution) for 1–2 h at room temperature and then washed as described above. The blots were exposed using Amersham Enhanced Chemiluminescence (ECL) Prime Western Blotting Detection Reagent (GE Healthcare). Image J analysis software was used to quantitatively analyze the bands. Glyceraldehyde 3-phosphate dehydrogenase (GAPDH) was used as a loading control (Santa Cruz Biotechnology, 1:1000 dilution).

### Quantitative real-time polymerase chain reaction (qPCR)

The total RNA from the cells was extracted using the TRIzol reagent (Invitrogen, CA, USA) according to the manufacturer’s protocol. A UV spectrophotometer was used to measure the purity and concentration of the RNA, and agarose gel electrophoresis was used to verify the RNA integrity. The cDNA templates were synthesized using the PrimeScript™ RT reagent kit with gDNA Eraser (TaKaRa, Japan) according to the manufacturer’s instructions. The qPCR analysis was performed using IQ SYBR Green Supermix (Bio-Rad, USA) and the Bio-Rad MJ Mini Opticon Real-Time PCR System, along with the Bio-Rad CFX Manager analysis software. The resulting amplification and melting curves were analyzed to identify the specific PCR products. The relative gene expression values were calculated using the comparative 2^–ΔΔCt^ method. The relative gene expression levels were calculated by normalization to the GAPDH mRNA level, whereas the miR-193a expression level was calculated by normalization to the U6 small nuclear RNA levels. The primer sequences were as follows: mouse GAPDH primers: sense: 5′-ACAACTTTGGCATTGTGGAA-3′, antisense: 5′-GATGCAGGGATGATGTTCTG-3′; mouse DNA methyltransferase 1 (DNMT1) primers: sense: 5′-CTGCTGTGGAGAAACTGGAA-3′, antisense: 5′-TGATTTCCGCCTCAATGATA-3′; mouse DNMT3α primers: sense: 5′-CTGTCCCATCCAGGCAGTAT-3′, antisense: 5′- CTTAGCGGTGTCTTGGAAGC-3′; mouse DNMT3β primers: sense: 5′-CTCAAACCCAACAAGAAGCA-3′, antisense: 5′-AGCAGCAGAGTCATTGGTTG-3′; mouse miR-193a primers: sense: 5′- AGGGTCTTTCCGCCCAGATTGTGAG -3′, antisense: 5′- AAGCTGCGGGAACATTGGGG-3.

### Bisulfite sequencing analysis

Genomic DNA was extracted from the cultured cells using a Qiagen ampR DNA Mini Kit (Qiagen Sciences, USA) according to the manufacturer’s recommended protocols. The concentration and purity of the DNA were measured by determining the absorbance at 260 and 280 nm. One microgram of genomic DNA from each sample was modified with sodium bisulfite using an EZ-96 DNA methylation kit (Zymo Research). The primers used for sodium bisulfite DNA sequencing analysis were designed using the online tool METHPRIMER: sense: 5'-GTTTTAGGTTTTAGATGGGGAAGTT-3', antisense: 5'-CTAAAATACCTTCCTCCCAATCAC-3'. The PCR products were sub-cloned and sequenced.

### Luciferase reporter assay

For the 3′-UTR-Renilla luciferase reporter assay, 293 T cells were seeded in 24-well plates at a density of 5.0 × 10^4^ cells/well and co-transfected with a reporter construct (psiCHECK2-ache-3′-UTR-wt or psiCHECK2-ache-3′-UTR-mut) and a miR-193a expression plasmid using the FuGENE HD transfection reagent (Roche Applied Science, Indianapolis, IN, USA) [14]. The cells were harvested 48 h after transfection. Luciferase activity was measured using the Dual Luciferase Reporter Assay System (Promega, USA) according to the manufacturer’s instructions.

### Infection with miR-193a lentivirus

The lentiviral pGIPZ plasmid expressing miR-193a and a negative control miRNA were designed and synthesized by Genechem (Shanghai, China) and named pLV-micro-RNA193a and pLV-miR-Ctrl, respectively. Viruses were harvested using Lenti-X Concentrator (Clontech) and stored at –80 °C. Primary c-kit-positive CSCs were infected with a lentivirus expressing pLVmicro-RNA193a or pLV-miR-Ctrl, and the infection efficiency was determined by measuring the expression of green fluorescent protein (GFP) using a fluorescence microscope (Additional file 2: Figure S1) and a qPCR analysis of miR-193a expression in infected cells (Additional file 2: Figure S2).

### Statistical analysis

The data were analyzed using SPSS software (v 11.5, SPSS Inc.). The results are presented as the mean ± standard deviation (SD) from at least three independent experiments, unless stated otherwise. Statistical analyses of the differences between two groups were performed using Student’s two-tailed *t* test, and one-way analysis of variance (ANOVA) was employed for comparisons between three groups. Statistical significance was defined as *P* < 0.05.

## Results

### Phenotypic characterization of CSCs and IGF-1-induced CSC migration and proliferation

**Fig. 1:**
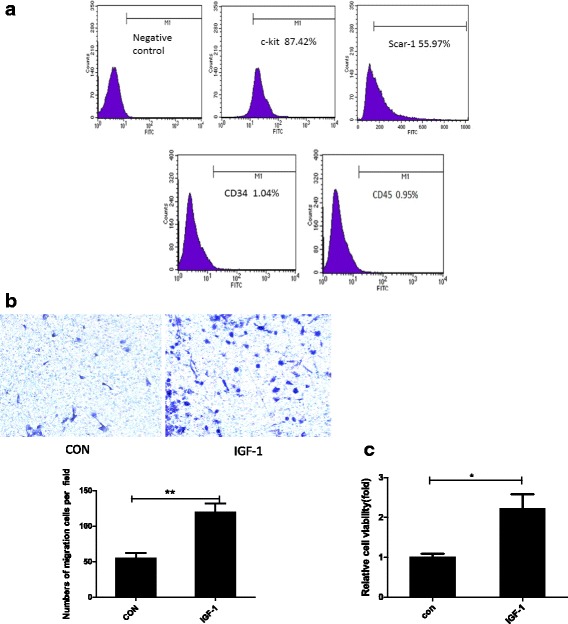
Phenotypic characterization of CSCs and IGF-1-induced CSC migration and proliferation. **a** Cells were identified by flow cytometry analysis of the following cell surface markers: c-kit (87.42%), Sca-1 (55.97%), CD34 (1.04%), and CD45 (0.95%). **b** Representative images of migrated CSCs at 48 h (stained with crystal violet) are shown (×100 magnification). **c** CSCs were stimulated with 100 ng/ml insulin-like growth factor-1 (IGF-1) for 72 h. A CCK-8 assay was used to analyze cell proliferation. The data were obtained from at least three independent experiments and are expressed as the mean ± SD. *P < 0.05, **P < 0.01, IGF-1 versus the control (CON) group

CSCs were harvested from 2-month-old C57BL/6 mouse hearts using gentle enzymatic digestion according to a previously described method [16]. C-kit magnetic beads were used to isolate CSCs by MACS. The CSCs were characterized by flow cytometry using the following cell surface markers: c-kit (87.42%), Sca-1 (55.97%), CD34 (1.04%), and CD45 (0.95%) (Fig. 1a). C-kit-positive CSCs were divided into two groups, the control group and the IGF-1 group (treated with 100 ng/ml IGF-1 for 72 h), to determine the effects of IGF-1 on CSC migration and proliferation. Based on the results of a Transwell migration experiment, a significantly larger number of c-kit-positive CSCs had migrated in the IGF-1 group (119.70 ± 21.55 cells per field) than in the control group (55.00 ± 12.77 cells per field) (Fig. 1b; *n* = 3, means ± SD; *P* < 0.05). Additionally, according to the results of the in vitro CCK-8 assays, the c-kit-positive CSCs in the IGF-1 group exhibited markedly higher proliferation rates than the cells in the control group (2.213 ± 0.735 OD vs. 1 ± 0.184 OD) (Fig. 1c; *n* = 3, means ± SD; *P* < 0.05). The IGF-1 group also showed higher proliferation rates than the control group using a BrdU method (Additional file 1: Figure S2-2). TUNEL indicate that IGF-1-mediated effects on cell apoptosis were no different compared with the control group (Additional file 1: Figure S2-2). Therefore, IGF-1 regulated CSC proliferation and migration.

### IGF-1 increased c-kit expression

**Fig. 2:**
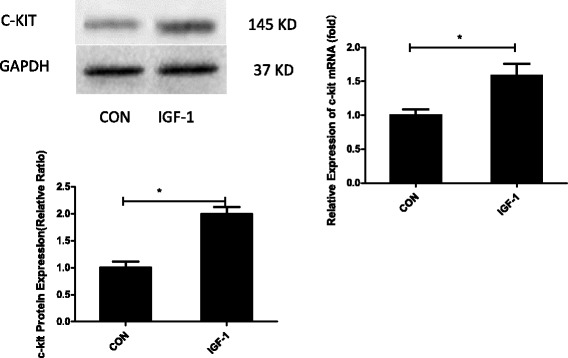
IGF-1 increased c-kit expression. CSCs were stimulated with 100 ng/ml insulin-like growth factor-1 (IGF-1) for 72 h. Western blots show that IGF-1 upregulates the expression of c-kit protein. qPCR results show that IGF-1 upregulates the expression of the c-kit mRNA. *P < 0.05, IGF-1 versus the control (CON) group

C-kit, also known as CD117, is a well-known stem cell marker. Because c-kit has been shown to be required for cell migration, proliferation, and survival in different models [9, 17, 18], we reasonably hypothesized that IGF-1 promotes the migration and proliferation of c-kit-positive CSCs by upregulating the c-kit expression level. Therefore, c-kit-positive CSCs were divided into two groups: the control group and the IGF-1 group (treated with 100 ng/ml IGF-1 for 72 h). These cells were analyzed by Western blotting and qPCR to determine the c-kit protein and mRNA level, and IGF-1 upregulated c-kit expression (Fig. 2) (*P* < 0.01).

### The PI3K/AKT pathway mediates the chemotactic and pro-growth effects of IGF-1 on c-kit-positive CSCs

**Fig. 3:**
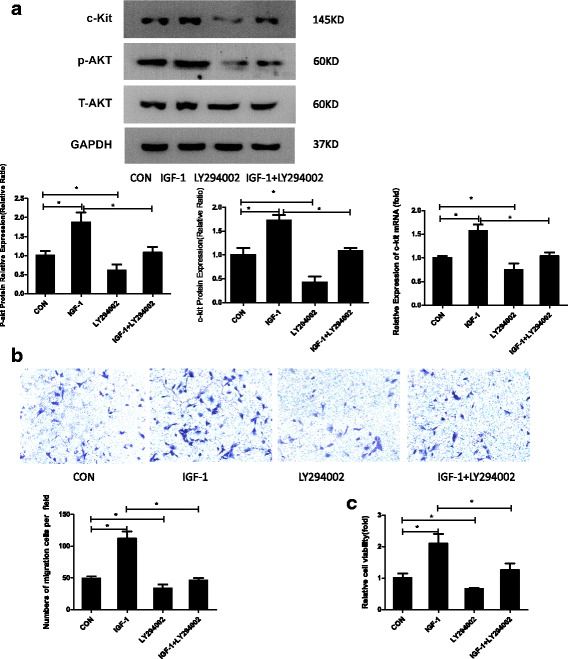
The PI3K/AKT pathway mediated the chemotactic and pro-growth effects of IGF-1 on c-kit-positive CSCs. **a** CSCs treated with PBS or 100 ng/ml insulin-like growth factor-1 (IGF-1) for 72 h, treated with 50 μM LY294002, or pretreated with 50 μM LY294002 for 30 min and then stimulated with 100 ng/ml IGF-1 for 72 h. Western blots show that IGF-1 upregulates the expression of the p-AKT and c-kit proteins compared with the control (CON) group (*P* < 0.05), and this effect is blocked by LY294002 (*P* < 0.05). The LY294002 group exhibited reduced p-AKT and c-kit expression (*P* < 0.05). q-PCR analysis of c-kit expression. The data were obtained from three independent experiments and are expressed as the mean ± SD; *n* = 3. *P < 0.05, between groups indicated. **b** Representative images of migrated CPCs at 48 h (stained with crystal violet) are shown (×100 magnification). The data were obtained from three independent experiments and are expressed as the means ± SD; *n* = 3. *P < 0.05, between groups indicated. **c** A CCK-8 assay was used to analyze cell proliferation. The data were obtained from at least three independent experiments and are expressed as the means ± SD. *P < 0.05, between groups indicated

The PI3K/AKT signaling pathway, a critical pathway downstream of IGF-1, is responsible for enhancing the proliferation of different kinds of stem cells, as well as increasing the migration of some cancer cells [16]. C-kit-positive CSCs were divided into four groups, a control group, pure IGF-1 group, LY294002 group, and IGF-1 + LY294002 group, to explore the molecular mechanism by which IGF-1 promotes c-kit expression in CSCs. Western blots were used to analyze the status of the PI3K/AKT signaling pathway and c-kit expression. As shown in Fig. 3a, the levels of the phosphorylated AKT and c-kit proteins were elevated in the IGF-1-treated group. In contrast, the IGF-1-mediated increase in the expression of phosphorylated AKT and c-kit was reversed by the PI3K-specific inhibitor LY294002 (Fig. 3a; *P* < 0.05). Furthermore, the LY294002 group exhibited altered expression of phosphorylated AKT and c-kit; the difference was significant compared with the control group (*P* < 0.05). Additionally, the IGF-1-mediated increase in c-kit expression was reversed by LY294002 in the qPCR experiment. Based on the results of a Transwell migration experiment, a significantly larger number of c-kit-positive CSCs had migrated in the pure IGF-1 group than in the control group (Fig. 3b; *n* = 3, mean ± SD; *P* < 0.05), and the effect was reversed by the PI3K/AKT signaling inhibitor LY294002 in the IGF-1 + LY294002 group compared with the IGF-1-treated group (*P* < 0.05). The migration of c-kit-positive CSCs in the LY294002 group showed a decreasing trend compared with the control group (*P* < 0.05). Additionally, according to the results of the CCK-8 assays in vitro, the proliferation rates of the c-kit-positive CSCs in the IGF-1 group were much higher than in the control group (Fig. 3c; *n* = 3, mean ± SD; *P* < 0.05). Similarly, this effect was also reversed by the PI3K/AKT signaling inhibitor LY294002 in the IGF-1 + LY294002 group (*P* < 0.05). Moreover, the LY294002 group showed altered migration compared with the control group (*P* < 0.05). Based on these findings, the PI3K/AKT pathway plays a pivotal role in the chemotactic and pro-growth effects of IGF-1 on c-kit-positive CSCs.

### IGF-1 upregulates DNMT expression via the PI3K/AKT pathway

**Fig. 4:**
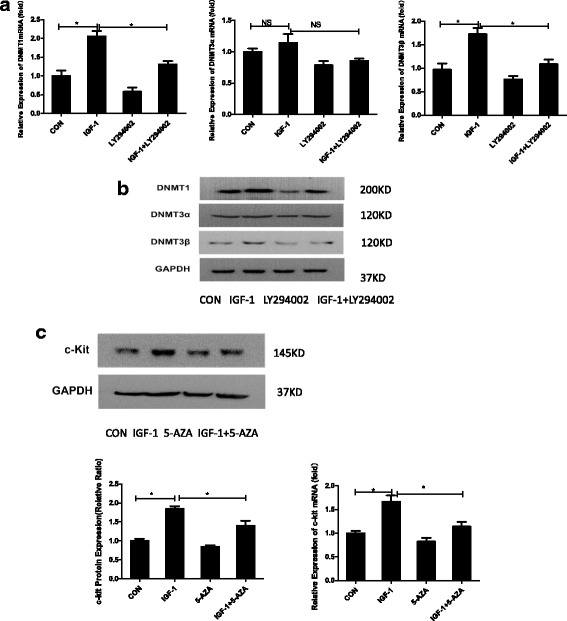
IGF-1 upregulated DNMTs via the PI3K/AKT pathway. **a** qPCR analysis of DNA methyltransferase (DNMT)1, DNMT3α, and DNMT3β expression in CSCs treated with PBS or 100 ng/ml insulin-like growth factor-1 (IGF-1) for 72 h, pretreated with 50 μM LY294002 for 30 min and then stimulated with 100 ng/ml IGF-1 for 72 h or treated with 50 μM LY294002. GAPDH expression was measured as a control (CON). The data were obtained from at least three independent experiments and are expressed as the mean ± SD. **b** Western blot analysis of DNMT1, DNMT3α, and DNMT3β expression in CSCs treated as described above. GAPDH expression was measured as a control. **c** CSCs were divided into four groups: normal control (CON), 100 ng/ml IGF-1 for 72 h, 1 μM 5-azacytidine (5-AZA) for 72 h, and both IGF-1 and 5-AZA. Western blots were performed to analyze the expression of the c-kit protein. q-PCR analysis of c-kit expression. The data were obtained from three independent experiments and expressed as the mean ± SD; *n* = 3. *P < 0.05, between groups indicated. NS not significant

We next explored the downstream effectors of the activated PI3K/AKT signaling pathway that contributed to the enhanced c-kit expression. The PI3K/AKT signaling pathway has been reported to regulate DNMTs such as DNMT1 and DNMT3b at both the RNA and protein level in cancer cells [19–21]. Based on our previous findings, DNMTs, particularly DNMT1 and DNMT3, are involved in regulating the c-kit protein level. C-kit-positive CSCs were divided into four groups as described above to verify our hypothesis, and Western blotting and qPCR (Fig. 4a, b) revealed that the expression of the DNMT1 and DNMT3β mRNAs and proteins was significantly upregulated in the IGF-1-treated group compared with the control group (*P* < 0.05), whereas the expression of the DNMT3α protein was similar in both groups (*P* > 0.05). Moreover, the increased expression of DNMT1 and DNMT3 was reversed when LY294002 was administered 30 min before the IGF-1 treatment compared with the pure IGF-1-treated group (*P* < 0.05). Furthermore, the effects of 5-azacytidine (5-AZA), a well-known inhibitor of DNA synthesis, on c-kit expression were assessed, as shown in Fig. 4c. 5-AZA clearly inhibited the catalytic activity of DNMTs in the 5-AZA + IGF-1 group compared with the control group and the IGF-1 group, respectively. Based on the results of the qPCR and Western blot assays, 5-AZA blocked the IGF-1-mediated increase in c-kit expression at both the mRNA and protein levels (Fig. 4c; *P* < 0.05). Thus, IGF-1 might upregulate c-kit expression via a PI3K/AKT/DNMT-dependent mechanism.

### miR-193a expression was downregulated in the IGF-1-stimulated group and negatively correlated with c-kit expression

**Fig. 5:**
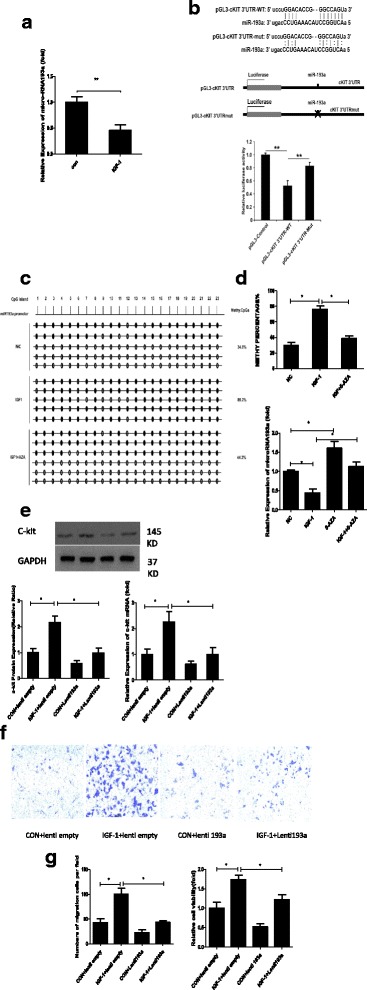
miR-193a expression was downregulated in the IGF-1-stimulated group and negatively correlated with c-kit expression. **a** qPCR was performed to analyze the expression of miR-193a. The data were obtained from three independent experiments and are expressed as the mean ± SD; *n* = 3. **P < 0.01, versus the control (CON) group. **b** The putative c-kit binding sequence GAGCA was mutated to CUCGU. The ‘x’ indicates the deleted miRNA-binding site, WT represents the wild-type sequence, and mut represents the mutant sequence. The graph below shows the normalized mean luciferase activity values of cells transfected with the miRNA expression plasmid compared with cells transfected with the empty vector pcDNA3.0; the luciferase activity of cells transfected with the pcDNA3.0 control plasmid was set to 1.0. The bars represent the SD. **P < 0.01. **c** CSCs were divided into the following groups: normal controls (NC), 100 ng/ml insulin-like growth factor-1 (IGF-1) for 72 h, 1 μM 5-azacytidine (5-AZA) for 72 h, and both IGF-1 and 5-AZA. qPCR was performed to analyze miR-193a expression. The data were obtained from three independent experiments and are expressed as the mean ± SD. *P < 0.05, between groups indicated. **d** Bisulfite pyrosequencing analysis of the DNA methylation status of the miR-193a promoter region in c-kit positive CSCs. **e** CSCs were infected with a control lentivirus or a lentivirus expressing miR-193a and stimulated with PBS or 100 ng/ml IGF-1 for 72 h. Western blots were performed to analyze the expression of the c-kit and GAPDH proteins. The data were obtained from at least three independent experiments and are expressed as the mean ± SD. *P < 0.05, between groups indicated. **f** Representative images of migrated CPCs at 48 h (stained with crystal violet) are shown (×100 magnification). The data were obtained from three independent experiments and are expressed as the mean ± SD; *n* = 3. *P < 0.05, between groups indicated. **g** CCK-8 assay of c-kit-positive CSCs from the different groups described above. The data were obtained from three independent experiments and are expressed as the mean ± SD; *n* = 3. *P < 0.05, versus the control group

In general, DNMTs negatively regulate the expression of downstream genes. However, DNMT activity was positively correlated with c-kit expression. Thus, we reasonably hypothesized that an important negative regulator of c-kit expression was controlled by DNMTs. In previous studies, miR-193a has been shown to be embedded in a CpG island and epigenetically repressed by promoter hypermethylation in leukemia cell lines, primary acute myeloid leukemia (AML) blasts, and ovarian cancer stem cells [14, 22]. Furthermore, the miR-193a level was reported to be negatively correlated with c-kit levels in these cells [17]. Hence, we measured miR-193a expression in cells with or without IGF-1 treatment. Based on the qPCR results, the IGF-1 treatment downregulated the expression of miR-193a compared with the control group (Fig. 5a; *P* < 0.01). Subsequently, luciferase activity was measured in 293 T cells co-transfected with a reporter construct (psiCHECK2-ache-3′-UTR-wt or psiCHECK2-ache-3′-UTR-mut) and a miR-193a expression plasmid. Micro-RNA193a hybridized with its predicted binding sites in the c-kit 3′-UTR (Fig. 5b; *P* < 0.01). We analyzed the methylation status of CpGs in the has-miR-193a promoter c-kit-positive CSC control group or the group treated with IGF-1 using bisulfite genome sequencing. As expected, the IGF-1 group showed a higher level of CpG methylation in the has-miR-193a promoter region compared with the control group, and this effect was partially blocked by 5-AZA (Fig. 5c). Isolated CSCs were treated with IGF-1, IGF-1 + 5-AZA, or 5-AZA alone to examine whether DNMTs regulated miR-193a expression. According to the results of the qPCR assay, miR-193a expression was upregulated in the IGF-1 + 5-AZA group compared with the IGF-1 group (Fig. 5d; *P* < 0.05). Subsequently, CSCs were infected with a control lentivirus or a lentivirus carrying miR-193a with or without IGF-1 stimulation. Following PBS administration, c-kit expression was comparable between CSCs infected with the two viruses. However, the IGF-1-induced increase in c-kit expression was significantly blunted by miR-193a overexpression (Fig. 5e; *P* < 0.05). In addition, IGF-1-mediated CSC migration and proliferation were also inhibited by miR-193a overexpression (Fig. 5f, g; *P* < 0.05). The expression of miR-193a was downregulated in the IGF-1-stimulated group and negatively correlated with c-kit expression.

## Discussion

C-kit-positive CSCs are a promising strategy for improving the environment within infarcted heart areas to enhance the quality of life in patients with CVD. A better understanding of the factors that promote c-kit-positive CSC migration, proliferation, and survival would help overcome the limitations of CSC therapy and provide promising targets for the treatment of CVD. Based on the findings of the present study, IGF-1 upregulated c-kit expression through a PI3K/AKT/DNMT pathway-dependent mechanism. Specifically, IGF-1 promoted the activation of PI3K/AKT signaling in CSCs by inducing AKT phosphorylation, resulting in DNMT activation; these effects were reversed by LY294002. Activated DNMTs then repressed miR-193a expression through promoter hypermethylation. In addition, miR-193a targeted the 3′-UTR of the c-kit mRNA to downregulate c-kit expression in c-kit-positive mouse CSCs. These results elucidate the molecular mechanism underlying the positive regulatory effects of IGF-1 on c-kit expression which plays an important role in c-kit-positive CSC migration and proliferation.

C-kit-positive CSCs were first separated and identified in a rodent model in 2003 [18] and were then viewed as a regenerative cell type that was able to differentiate into all cardiac lineages, including cardiomyocytes, endothelial cells, and smooth muscle cells. Therefore, over the past two decades, c-kit-positive CSCs have been implanted into different animal models, and have shown promising results in treating left ventricular dysfunction, heart failure, and chronic ischemic cardiomyopathy [23–25]. In clinical studies [24], c-kit-positive CSCs exerted beneficial effects on left ventricular function, improved patient quality of life, and reduced the infarct size. However, based on experiments from different laboratories [26, 27], controversy exists regarding the ability of minimally c-kit-positive CSCs to differentiate into cardiomyocytes. Notably, the differentiation of c-kit-positive CSCs into non-cardiomyocytes and their paracrine effects may play an important role in cell therapy [25, 27–29]. Therefore, the study of the migration and proliferation of c-kit-positive CSCs has far-reaching significance.

As shown in our previous investigation, increased c-kit expression enhanced the proliferation and migration of c-kit-positive CSCs in mice [30]. Consistent with these findings, c-kit activation increased the growth and survival of human c-kit-positive CSCs and reduced CSC apoptosis under serum starvation or even oxidative stress conditions. During these processes, both the PI3K/AKT and MEK/ERK pathways were responsible for the effects of c-kit [6]. The present study focused on molecules downstream of the PI3K/AKT pathway, which clarified the mechanism underlying the pro-survival effects of IGF-1 on c-kit-positive CSCs. Furthermore, IGF-1-mediated activation of the PI3K/AKT pathway enhanced CSC migration and proliferation by upregulating c-kit expression.

AKT stabilizes DNMT1 by phosphorylating Ser 143, and the dephosphorylation of this site makes DNMT1 vulnerable to ubiquitin-mediated proteasomal degradation [31]. In multiple human cell lines, such as bladder cancer and prostate cancer cells, levels of the DNMT1 and DNMT3β proteins are modulated by the PI3K/AKT/glycogen synthase kinase 3β (GSK3β) signaling pathway. Specifically, activated PI3K/AKT stabilizes the DNMT1 protein by inhibiting the ubiquitin/proteasome-mediated degradation of DNMT1 [19, 21]. Consistent with previous reports, the expression of DNMT1 and DNMT3β in c-kit-positive CSCs was significantly upregulated by IGF-1-induced PI3K/AKT activation in our study. Additionally, a rescue experiment using 5-AZA, a specific inhibitor of DNMT activity, revealed that elevated DNMT protein levels are required for IGF-1-induced c-kit upregulation.

DNA methylation, an epigenetic modification that occurs at CpG islands, is considered a vital mechanism regulating gene expression. According to several studies, miR-193a is embedded in a CpG island, and hypermethylation of the miR-193a promoter results in its epigenetic silencing in AML [14, 20], non-small cell lung cancer [32], and ovarian cancer [22]. Although the role of miR-193a in c-kit-positive CSCs was unclear, we hypothesized that IGF-1/PI3K/AKT-induced DNMT activation would epigenetically silence miR-193a expression, eventually leading to c-kit upregulation in CSCs. This hypothesis was validated by our experiments.

This study elucidates the underlying mechanism by which IGF-1 upregulates c-kit expression to promote the migration and proliferation of c-kit-positive resident CSCs. Our study is the first to show that miR-193a negatively changed c-kit expression is modulated by DNMT activity. In our study, the expression of miR-193a and c-kit was decreased by infection with a miR-193-expressing lentivirus or 5-AZA treatment. Moreover, miR-193a expression was altered following the inhibition of the PI3K/AKT pathway. This finding differs from a report showing that forkhead box O3a (FoxO3a) altered the expression of key downstream regulators of the cell cycle in c-kit-positive CSCs [9]. Our study focused on the epigenetic regulation of gene expression. We revealed the involvement of DNA methylation and the role of miR-193a in regulating gene expression, and the results verify that IGF-1 improved the proliferation of c-kit-positive CSCs as a potential treatment for cardiac regeneration. These findings suggest approaches for maintaining the physiological characteristics of c-kit-positive CSCs and overcoming their limited migration and proliferation in the future.

However, the mechanism of c-kit modulation is quite complicated and may be linked with other cell signaling pathways (such as PTEN) [33]. Recently, some specific microRNAs were reported to mediate the regulation of DNMT modulation; miR-29b repressed DNMT3α and DNMT3β expression directly and DNMT1 indirectly in AML [34]. While our present study only focused downstream of DNMTs, we propose to further study in the future how DNMTs are regulated by IGF-1. In addition, other microRNAs (miR-137, miR-218) may bind to the 3’-UTR of c-kit mRNA and modify the c-kit expression level. These problems should be addressed in future studies.

## Conclusions

IGF-1 upregulated c-kit expression in c-kit-positive CSCs, resulting in enhanced CSC proliferation and migration, by activating the PI3K/AKT/DNMT signaling pathway to epigenetically silence miR-193a, which negatively modifies the c-kit expression level.

## Additional files

Additional file 1: Supplementary data. (DOCX 1754 kb)

Additional file 2: **Figure S1.** Flow cytometry analysis of an efficiency of c-kit-positive CSCs transfected with lenti-193a. Percentage of FAM-positive cells at 48 h after transfection is indicated, lenti-empty used as control. **Figure S2.** qPCR analysis showing the upregulation of miR193a in infected cells (3.14-fold) compared with control group. (PPTX 155 kb)

## Abbreviations

5-AZA: 5-Azacytidine
AML: Acute myeloid leukemia
CCK-8: Cell Counting Kit-8
CSC: Cardiac stem cell
CVD: Cardiovascular disease
DNMT: DNA methyltransferase
GAPDH: Glyceraldehyde-3-phosphate dehydrogenase
IGF: Insulin-like growth factor
MACS: Magnetic-activated cell sorting
OD: Optical density
PBS: Phosphate-buffered saline
PI3K: Phosphoinositol 3-kinase
qPCR: Quantitative real-time polymerase chain reaction

## References

[1] Mozaffarian D, Benjamin EJ, Go AS, Arnett DK, Blaha MJ, Cushman M, Das SR, de Ferranti S, Despres JP, Fullerton HJ (2016). Heart disease and stroke statistics—2016 update: a report from the American Heart Association. Circulation.

[2] Kelly BB, Narula J, Fuster V (2012). Recognizing global burden of cardiovascular disease and related chronic diseases. Mt Sinai J Med.

[3] Linke A, Muller P, Nurzynska D, Casarsa C, Torella D, Nascimbene A, Castaldo C, Cascapera S, Bohm M, Quaini F (2005). Stem cells in the dog heart are self-renewing, clonogenic, and multipotent and regenerate infarcted myocardium, improving cardiac function. Proc Natl Acad Sci U S A.

[4] Ellison GM, Vicinanza C, Smith AJ, Aquila I, Leone A, Waring CD, Henning BJ, Stirparo GG, Papait R, Scarfo M (2013). Adult c-kit(pos) cardiac stem cells are necessary and sufficient for functional cardiac regeneration and repair. Cell.

[5] Molkentin JD, Houser SR (2013). Are resident c-kit + cardiac stem cells really all that are needed to mend a broken heart?. Circ Res.

[6] Vajravelu BN, Hong KU, Al-Maqtari T, Cao P, Keith MCL, Wysoczynski M, Zhao J, Moore JB, Bolli R (2015). C-kit promotes growth and migration of human cardiac progenitor cells via the PI3K-AKT and MEK-ERK pathways. PLoS One.

[7] Ye P, D'Ercole AJ (2006). Insulin-like growth factor actions during development of neural stem cells and progenitors in the central nervous system. J Neurosci Res.

[8] D'Amario D, Cabral-Da-Silva MC, Zheng H, Fiorini C, Goichberg P, Steadman E, Ferreira-Martins J, Sanada F, Piccoli M, Cappetta D (2011). Insulin-like growth factor-1 receptor identifies a pool of human cardiac stem cells with superior therapeutic potential for myocardial regeneration. Circ Res.

[9] Johnson AM, Kartha CC (2014). Proliferation of murine c-kitpos cardiac stem cells stimulated with IGF-1 is associated with Akt-1 mediated phosphorylation and nuclear export of FoxO3a and its effect on downstream cell cycle regulators. Growth Factors.

[10] Bagno LL, Carvalho D, Mesquita F, Louzada RA, Andrade B, Kasai-Brunswick TH, Lago VM, Suhet G, Cipitelli D, Werneck-de-Castro JP (2016). Sustained IGF-1 secretion by adipose-derived stem cells improves infarcted heart function. Cell Transplant.

[11] Bartel DP (2004). MicroRNAs: genomics, biogenesis, mechanism, and function. Cell.

[12] Haetscher N, Feuermann Y, Wingert S, Rehage M, Thalheimer FB, Weiser C, Bohnenberger H, Jung K, Schroeder T, Serve H (2015). STAT5-regulated microRNA-193b controls haematopoietic stem and progenitor cell expansion by modulating cytokine receptor signalling. Nat Commun.

[13] Li N, Long B, Han W, Yuan S, Wang K. microRNAs: important regulators of stem cells. Stem Cell Research & Therapy. 2017;8(1):110. 10.1186/s13287-017-0551-0 PMC5426004 28494789

[14] Gao XN, Lin J, Li YH, Gao L, Wang XR. MicroRNA-193a represses c-kit expression and functions as a methylation-silenced tumor suppressor in acute myeloid leukemia. Oncogene. 2011;30(31):3416–28. 10.1038/onc.2011.62 21399664

[15] Xu R, Sun Y, Chen Z, Yao Y, Ma G (2016). Hypoxic preconditioning inhibits hypoxia-induced apoptosis of cardiac progenitor cells via the PI3K/Akt-DNMT1-p53 pathway. Sci Rep.

[16] Wongtrakoongate P (2015). Epigenetic therapy of cancer stem and progenitor cells by targeting DNA methylation machineries. World J Stem Cells.

[17] Li Y, Gao L, Luo X, Wang L, Gao X, Wang W, Sun J, Dou L, Li J, Xu C (2013). Epigenetic silencing of microRNA-193a contributes to leukemogenesis in t(8;21) acute myeloid leukemia by activating the PTEN/PI3K signal pathway. Blood.

[18] Beltrami AP, Barlucchi L, Torella D, Baker M, Limana F, Chimenti S, Kasahara H, Rota M, Musso E, Urbanek K (2003). Adult cardiac stem cells are multipotent and support myocardial regeneration. Cell.

[19] Agarwal S, Amin KS, Jagadeesh S, Baishay G, Rao PG, Barua NC, Bhattacharya S, Banerjee PP (2013). Mahanine restores RASSF1A expression by down-regulating DNMT1 and DNMT3B in prostate cancer cells. Mol Cancer.

[20] Mei C, Sun L, Liu Y, Yang Y, Cai X, Liu M, Yao W, Wang C, Li X, Wang L (2010). Transcriptional and post-transcriptional control of DNA methyltransferase 3B is regulated by phosphatidylinositol 3 kinase/Akt pathway in human hepatocellular carcinoma cell lines. J Cell Biochem.

[21] Sun L, Zhao H, Xu Z, Liu Q, Liang Y, Wang L, Cai X, Zhang L, Hu L, Wang G (2007). Phosphatidylinositol 3-kinase/protein kinase B pathway stabilizes DNA methyltransferase I protein and maintains DNA methylation. Cell Signal.

[22] Cheng FH, Aguda BD, Tsai JC, Kochanczyk M, Lin JM, Chen GC, Lai HC, Nephew KP, Hwang TW, Chan MW (2014). A mathematical model of bimodal epigenetic control of miR-193a in ovarian cancer stem cells. PLoS One.

[23] Tang XL, Rokosh G, Sanganalmath SK, Yuan F, Sato H, Mu J, Dai S, Li C, Chen N, Peng Y (2010). Intracoronary administration of cardiac progenitor cells alleviates left ventricular dysfunction in rats with a 30-day-old infarction. Circulation.

[24] Sanganalmath SK, Bolli R (2013). Cell therapy for heart failure: a comprehensive overview of experimental and clinical studies, current challenges, and future directions. Circ Res.

[25] Bolli R, Tang XL, Sanganalmath SK, Rimoldi O, Mosna F, Abdel-Latif A, Jneid H, Rota M, Leri A, Kajstura J (2013). Intracoronary delivery of autologous cardiac stem cells improves cardiac function in a porcine model of chronic ischemic cardiomyopathy. Circulation.

[26] van Berlo JH, Kanisicak O, Maillet M, Vagnozzi RJ, Karch J, Lin SJ, Middleton RC, Marbán E, Molkentin JD (2014). c-kit + cells minimally contribute cardiomyocytes to the heart. Nature.

[27] Hong KU, Li QH, Guo Y, Patton NS, Moktar A, Bhatnagar A, Bolli R (2013). A highly sensitive and accurate method to quantify absolute numbers of c-kit + cardiac stem cells following transplantation in mice. Basic Res Cardiol.

[28] Kazakov A, Meier T, Werner C, Hall R, Klemmer B, Körbel C, Lammert F, Maack C, Böhm M, Laufs U (2015). C-kit + resident cardiac stem cells improve left ventricular fibrosis in pressure overload. Stem Cell Res.

[29] Ellison GM, Vicinanza C, Smith AJ, Aquila I, Leone A. Adult c-kitCardiac Stem Cells Are Necessary and Sufficient for Functional Cardiac Regeneration and Repair. Cell. 2013;154(4):827–42. 10.1016/j.cell.2013.07.039 23953114

[30] Chen Z, Pan X, Yao Y, Yan F, Chen L, Huang R, Ma G (2013). Epigenetic regulation of cardiac progenitor cells marker c-kit by stromal cell derived factor-1alpha. PLoS One.

[31] Esteve PO, Chang Y, Samaranayake M, Upadhyay AK, Horton JR, Feehery GR, Cheng X, Pradhan S (2011). A methylation and phosphorylation switch between an adjacent lysine and serine determines human DNMT1 stability. Nat Struct Mol Biol.

[32] Heller G, Weinzierl M, Noll C, Babinsky V, Ziegler B, Altenberger C, Minichsdorfer C, Lang G, Dome B, End-Pfutzenreuter A (2012). Genome-wide miRNA expression profiling identifies miR-9-3 and miR-193a as targets for DNA methylation in non-small cell lung cancers. Clin Cancer Res.

[33] Ma J, Sawai H, Matsuo Y, Ochi N, Yasuda A, Takahashi H, Wakasugi T, Funahashi H, Sato M, Takeyama H (2010). IGF-1 mediates PTEN suppression and enhances cell invasion and proliferation via activation of the IGF-1/PI3K/Akt Signaling pathway in pancreatic cancer cells. J Surg Res.

[34] Garzon R, Liu S, Fabbri M, Liu Z, Heaphy CEA, Callegari E, Schwind S, Pang J, Yu J, Muthusamy N (2009). MicroRNA-29b induces global DNA hypomethylation and tumor suppressor gene re-expression in acute myeloid leukemia by targeting directly DNMT3A and 3B and indirectly DNMT1. Blood.

