# Supplementary material for: Insulin-like growth factor-1-mediated regulation of miR-193a expression promotes the migration and proliferation of c-kit-positive mouse cardiac stem cells
Source: Stem Cell Res Ther. 2018 Feb 21;9:41. doi: 10.1186/s13287-017-0762-4 (PMC5822561; doi:10.1186/s13287-017-0762-4)
Supplement: Supplementary file 1 — Supplementary data. (DOCX 1754 kb) [file 13287_2017_762_MOESM1_ESM.docx]

**Supplementary Data**

**Insulin-like growth factor (IGF)-1-****mediated regulation of miR-193a expression promotes the migration and proliferation of c-kit-positive mouse cardiac stem cells**

Authors:

Yuning Sun^1^, Rongfeng Xu^1^, Jia Huang^1^, Yuyu Yao^1^, Xiaodong Pan^1^, Zhongpu Chen*, & Genshan Ma*

**Expended methods**

**5-Bromo-2′-deoxyuridine (BrdU) incorporation assay**

To assess cell proliferation, a 5-bromo-2′-deoxyuridine (BrdU) incorporation assay was used with the BrdU incorporation assay kit (Roche , USA). The cells were seeded at a density of 2×10^3^ cells per well with or without IGF-1 in 96-well microtiter plates. After 72 h, BrdU–labeling solution was added to the cells. After DNA denaturation, peroxidase-labeled anti-BrdU monoclonal antibody was added to the cells and the samples were incubated at room temperature for 90 min. The BrdU-antibody complexes were detected by luminometer. All samples used in the experiments contained 0.0003% DMSO; the presence of DMSO did not affect cell proliferation.

**Apoptosis analysis**

Apoptosis analysis was performed using the terminal deoxynucleotidyl transferase-mediated dUTP nick end-labeling (TUNEL, USA) method in accordance with the manufacturer's protocol. Cells were treated with or without IGF-1 for 72h. The cells were washed with PBS for 3 times and fixed with 4% paraformaldehyde for 20min at RT. The cells were treated with 0.1% triton X-100 solution for 2 min and incubated with 20 μl TUNEL reaction mixtures for 1 h at RT. The nucleus counterstaining was performed using DAPI (Invitrogen, CA, USA). The cells were imaged by fluorescence microscopy. The TUNEL-positive (red-fluorescing) cells were determined for an estimation of apoptosis. The rate of apoptosis in cells was calculated as the number of TUNEL-positive cells divided by total number of cells with DAPI stain.

**Supplementary Figures**

Figure S2-1

Figure S2-1 Cells were manually counted in three random fields before and after transwell experiments in the upper chambers using a phase contrast microscope .The data were obtained from three independent experiments and are expressed as the means±SD. IGF-1 group showed decreasing trend than the con group that means cells were migrated by IGF-1.

Figure S2-2

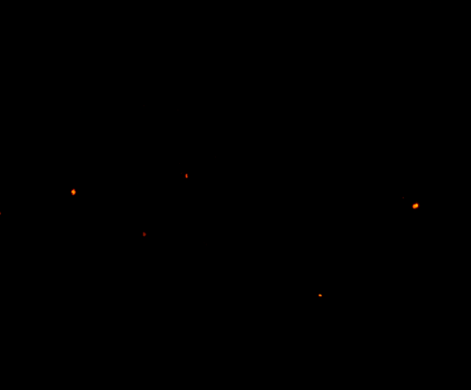

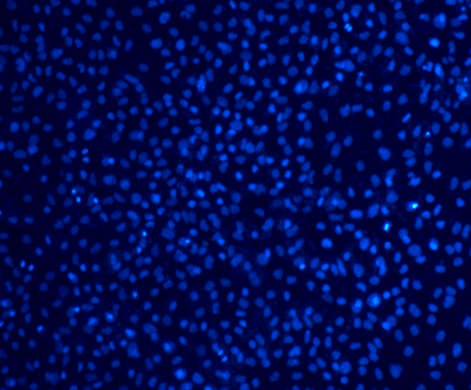

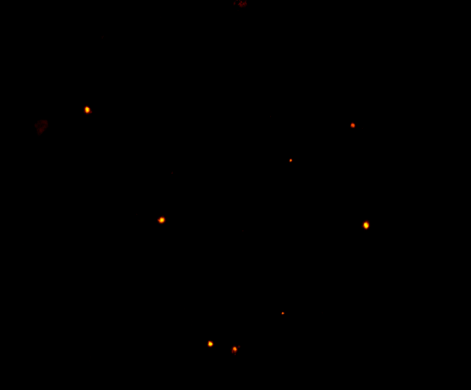

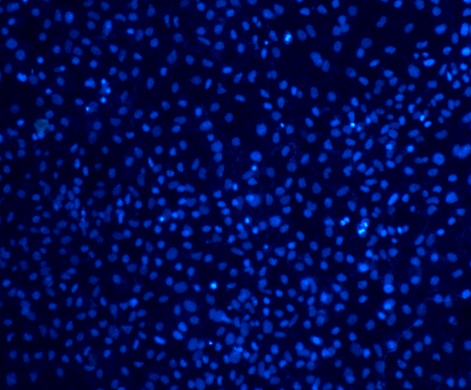


B

A

C

**Figure S2-2. Effects of IGF-1 on c-kit cardiac stem cells proliferation and apoptosis. (**A) Effect of IGF-1 on c-kit cardiac stem cells proliferation. Cells (2×10^3^) were incubated with or without IGF-1 in 96-well microplate. Cell proliferation was quantified using a BrdU method. Proliferation in the quiescent state was deemed to be 100% (*n* = 3). **P* < 0.05. (B) The percentage of apoptotic cells was determined by comparing the number of TUNEL-positive cells to total number of DAPI-stained cells. The response in the untreated cell was considered to be 100% (*n* = 3). (C) Representative photomicrographs. Scale bar: 400 μm. Green colors, TUNEL-positive cells; Blue colors, DAPI-stained cells.

Figure S3-1

Figure S3-1 qPCR was performed to analyze the expression of miR-193a. Experiments were divided into four groups: con, IGF-1, LY294002, IGF-1+LY294002. The data were obtained from three independent experiments and expressed as the means±SD. n=3. *P<0.05 IGF-1 group compared with the control group. *P<0.05 IGF-1 group compared with IGF-1+LY294002 group.

Figure S3-2

Con IGF-1 IGF-1+ I‑OMe AG538


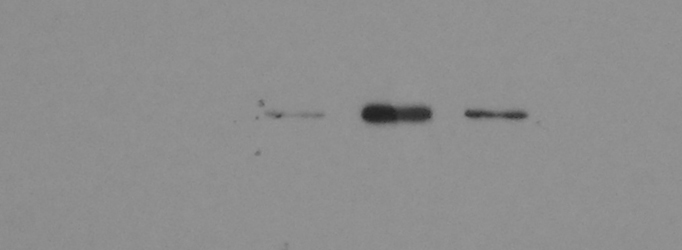

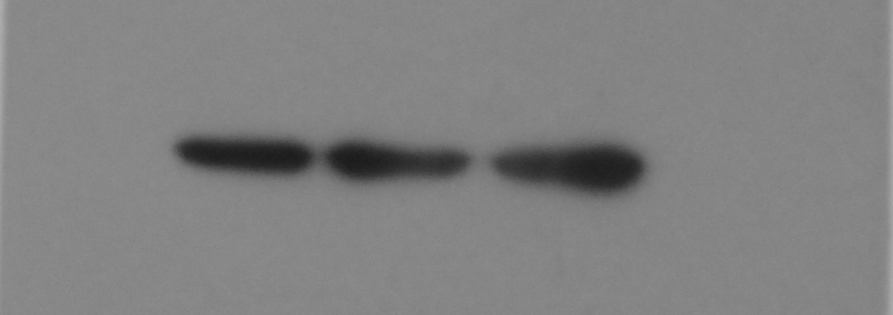


P-akt

T-akt

GAPDH

60KD

60KD

37KD


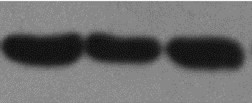


Figure S3-2 IGF-1 regulates PI3K/Akt pathway in c-kit cardiac stem cells. Starved cells were treated with 10 µmol/l  I-OMe AG538 for 3 h followed by stimulation with 100 ng/ml IGF-1 for 72h. T-Akt and p-Akt expressions were determined by western blot. Data represents the mean ± S.D. (*P < 0.05 versus control).
