# Supplementary figures and images for: Insulin-like growth factor-1-mediated regulation of miR-193a expression promotes the migration and proliferation of c-kit-positive mouse cardiac stem cells
Source: Stem Cell Res Ther. 2018 Feb 21;9:41. doi: 10.1186/s13287-017-0762-4 (PMC5822561; doi:10.1186/s13287-017-0762-4)

## Slide 1
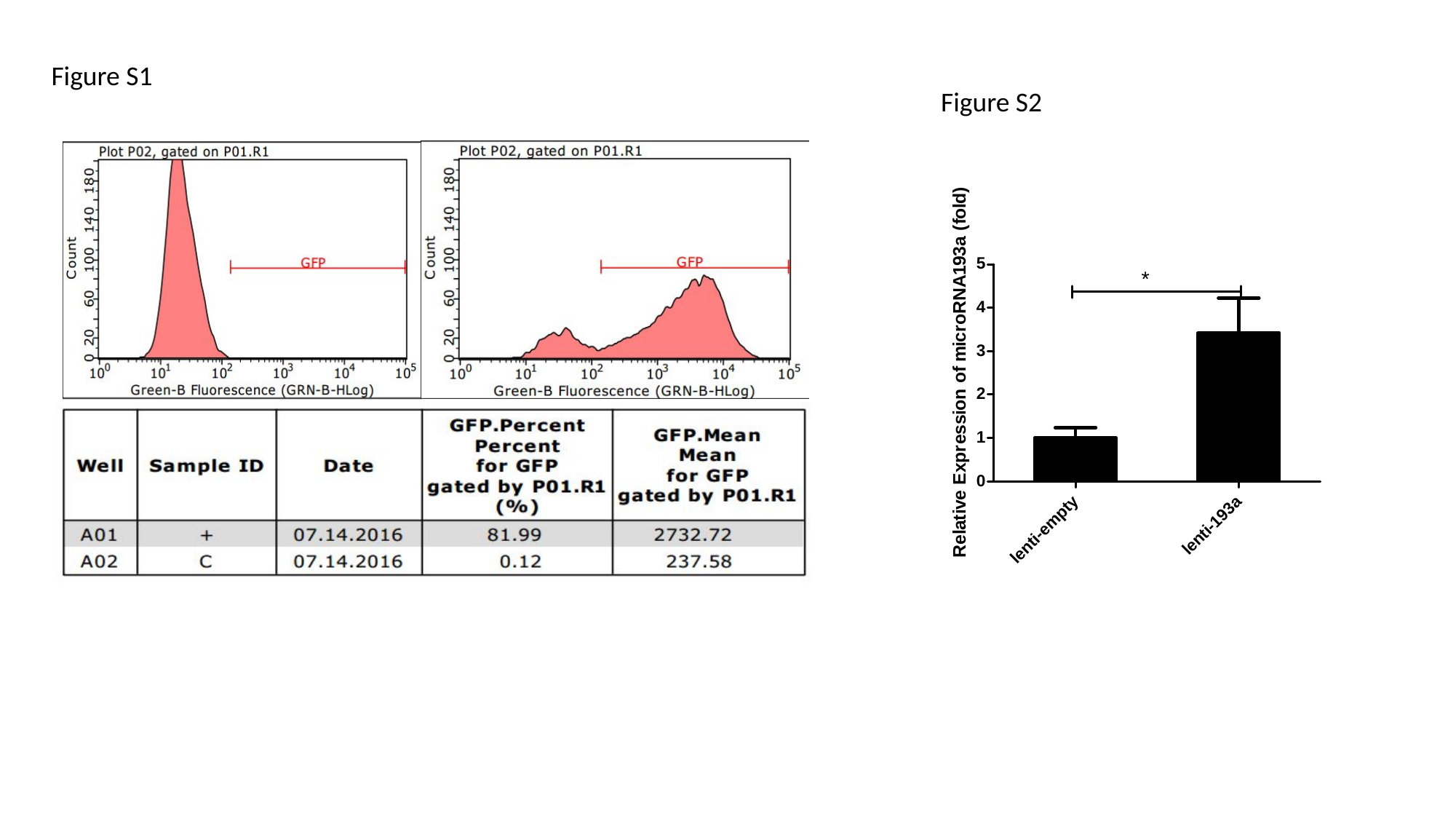

Figure S1
Figure S2

Supplement: Supplementary file 2 — Figure S1. Flow cytometry analysis of an efficiency of c-kit-positive CSCs transfected with lenti-193a. Percentage of FAM-positive cells at 48 h after transfection is indicated, lenti-empty used as control. Figure S2. qPCR analysis showing the upregulation of miR193a in infected cells (3.14-fold) compared with control group. (PPTX 155 kb) [file 13287_2017_762_MOESM2_ESM.pptx]
